# Biome‐ and timescale‐dependence of Holocene vegetation variability in the Northern Hemisphere

**DOI:** 10.1002/ece3.10585

**Published:** 2023-10-24

**Authors:** Raphaël Hébert, Laura Schild, Thomas Laepple, Ulrike Herzschuh

**Affiliations:** ^1^ Alfred Wegener Institute, Helmholtz Centre for Polar and Marine Research Potsdam Germany; ^2^ Institute of Geosciences, University of Potsdam Potsdam Germany; ^3^ MARUM – Center for Marine Environmental Sciences and Faculty of Geosciences University of Bremen Bremen Germany; ^4^ Institute of Biochemistry and Biology, University of Potsdam Potsdam Germany; ^5^ Institute of Environmental Science and Geography, University of Potsdam Potsdam Germany

**Keywords:** (de)stabilizing feedback, biome classification, climate‐vegetation link, Haar structure function, Holocene vegetation variability, pollen assemblages

## Abstract

Global climatic changes expected in the next centuries are likely to cause unparalleled vegetation disturbances, which in turn impact ecosystem services. To assess the significance of disturbances, it is necessary to characterize and understand typical natural vegetation variability on multi‐decadal timescales and longer. We investigate this in the Holocene vegetation by examining a taxonomically harmonized and temporally standardized global fossil pollen dataset. Using principal component analysis, we characterize the variability in pollen assemblages, which are a proxy for vegetation composition, and derive timescale‐dependent estimates of variability using the first‐order Haar structure function. We find, on average, increasing fluctuations in vegetation composition from centennial to millennial timescales, as well as spatially coherent patterns of variability. We further relate these variations to pairwise comparisons between biome classes based on vegetation composition. As such, higher variability is identified for open‐land vegetation compared to forests. This is consistent with the more active fire regimes of open‐land biomes fostering variability. Needleleaf forests are more variable than broadleaf forests on shorter (centennial) timescales, but the inverse is true on longer (millennial) timescales. This inversion could also be explained by the fire characteristics of the biomes as fire disturbances would increase vegetation variability on shorter timescales, but stabilize vegetation composition on longer timecales by preventing the migration of less fire‐adapted species.

## Graphical abstract

We investigate vegetation variability in the Holocene vegetation by examining a taxonomically harmonized and temporally standardized global fossil pollen dataset. Using principal component analysis, we characterize how vegetation compositions vary in the pollen assemblages and derive timescale‐dependent estimates of variability using the first‐order Haar structure function. We find, on average, increasing fluctuations in vegetation composition from centennial to millennial timescale, as well as spatially coherent patterns of variability, and further relate these variations to pairwise comparisons between biome classes based on vegetation composition.

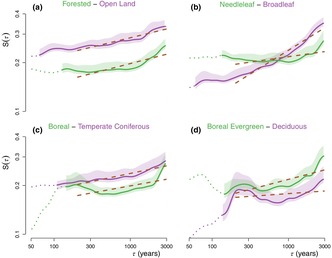

## 1 INTRODUCTION

Global climatic changes expected in the 21st century are likely to create unparalleled disturbances on vegetation (Diffenbaugh & Field, 2013). It is thus necessary to characterize and understand the typical natural vegetation variability in order to evaluate the significance of any future vegetation response and also improve management planning for climate change adaptation (Keane et al., 2009). Current restoration and management of forests also rely on the concept of natural variability in forests in order to establish the appropriate composition for a given area based on the range of known ecological and climatic conditions (Landres et al., 1999). It is challenging to study the “normal” natural variability of current forests given that the vast majority of forests have already been highly impacted or decimated by human activities (Angelstam & Kuuluvainen, 2004; Burley et al., 2004) and therefore studies of the variability in near‐natural forests can only rely on small spatial scales (Kuuluvainen, 2002), and are limited by short observational records. Vegetation dynamics do, however, take place on a vast range of spatial and temporal scales from localized gap dynamics (Yamamoto, 2000), to cohort dynamics and broad‐scale succession (Kuuluvainen, 2016). It is therefore necessary to investigate the paleorecord in order to study long‐term vegetation variability on large spatial scales.

Sub‐fossil pollen remains accumulated in peatlands, lake sediments and marine sediments have been used to reconstruct past vegetation (Prentice & Iii, 1998). Pollen grains are identified and counted to derive pollen assemblages, that is, the relative proportion of different pollen types present, which are indicators of past nearby vegetation composition. The long history of pollen statistics (von Post, 1946) has led to the compilation of thousands of records, providing us with a vegetation proxy with the most extensive spatio‐temporal coverage (Williams et al., 2018); there are however several challenges to derive systematic estimates of vegetation variability given the irregular sampling and varying temporal resolution of different pollen records (Hébert et al., 2021). Another challenge in integrating a large database of pollen records lies in the varying taxonomies between studies. Harmonization at a higher level allows for consistent large‐scale investigations (Cao et al., 2020), although this presents another challenge to distinguish biomes dominated by different species of the same taxa. Finally, the multivariate nature of pollen data requires appropriate methods to define a univariate measure of vegetation variability consistent across records.

While time series analysis methods are possible for multivariate data, they are more challenging and difficult to interpret (Beeram & Kuchibhotla, 2021). Therefore, it is often advantageous to reduce to univariate data in order to use more established methods (Legendre & Legendre, 2012). Principal components analysis (PCA) is a widely used method in ecology (Djamali et al., 2009; Festi et al., 2015; Tian et al., 2022; Wang et al., 2017; Zhao et al., 2012), which consists of rotating the coordinate system (taxa proportions in the case of pollen assemblages) in order to maximize the variance explained along fewer dimensions such that the newly rotated axes are linear combinations of the original axes. Alternatively, nonlinear methods such as nonmetric multidimensional scaling (Gavin, 2015; Goring et al., 2009; Julier et al., 2018; Kenkel & Orloci, 1986; Papadopoulou et al., 2022) or principal curves (De'ath, 1999; Herzschuh et al., 2016) have been used in ecology. While such methods can improve the description of the data in reduced dimensions, they rely on iterative approaches and require careful evaluation to avoid artefacts. This can be impractical when dealing with a large database of records and we thus prefer the simpler PCA as it can be more easily, and uniformly, applied and interpreted across all our sites.

Characterizing variability across timescales, using for example spectral analysis, is informative of the underlying characteristics of a time series such as the temporal autocorrelation and stationarity, and can therefore be used as a measure of stability for ecological communities. In addition, it can inform us on the underlying drivers and mechanisms of variability related to other elements such as the climate. Climate variability has been shown to occur over a vast range of timescales, using instrumental and paleoclimate data, with increasing variance from decadal to multi‐millennial timescales (Hébert et al., 2022; Huybers & Curry, 2006; Laepple & Huybers, 2014a; Schmitt et al., 1995; Yiou et al., 1996). While temporal variability of vegetation has been studied on daily to decadal timescales using satellite data (Brando et al., 2010; Lara et al., 2018; Sebastian et al., 2019; Claessen et al., 2017), large‐scale studies of past vegetation have focused on reconstructing the mean biomes for specific timeslices (Prentice & Jolly, 2000), and thus virtually nothing is known with respect to the statistics of vegetation variability on multi‐decadal timescales and longer beyond single site case studies.

Vegetation variability is the result of internal vegetation processes and external influences such as the climate. Vegetation and climate are generally thought to be in dynamical equilibrium on long enough timescales (Chevalier et al., 2020; Webb, 1986). As such, climate‐related variability is the main driver of millennial‐scale variability, whereas internal vegetation processes may play a relatively larger role on centennial timescales. Therefore, the scaling behaviour of variability can indicate the relative importance of the long‐term climate influence relative to internal vegetation processes. When the amplitude of fluctuations decreases with timescales, this means that the community composition converges towards a stable mean, which can be indicative of a similarly stable climate, or stabilizing feedback acting even on long timescales and allowing vegetation composition to maintain itself in a state of climate disequilibrium (Dallmeyer et al., 2022; Herzschuh et al., 2016; Scheffer et al., 2012). Alternatively, when the amplitude of fluctuations increases with timescale, this could be the result of a long‐term climate trend, and/or stabilizing feedback acting on shorter timescales. For example, forests have a significant impact on local climate through biogeophysical feedback, and the increased transpiration linked to forest presence leads to higher soil moisture and precipitation, thus forming a stabilizing feedback for forest presence (Bonan, 2008; Claussen, 1998).

In this study, we aim to produce consistent estimates of vegetation variability from centennial to millennial timescales using a large dataset of pollen records covering the northern hemisphere extra‐tropical regions (Herzschuh et al., 2022). Then, we relate them to vegetation composition in order to explain the observed spatial patterns. To this end, we develop a biome classification procedure based on modern biome and land‐cover classes combined with recent pollen samples. We then perform an iterative set of binary comparisons, starting with an ecologically broad comparison between forested and open‐land biomes, and then subsequently refining to more specific comparisons of broadleaf and needleleaf forest types, of temperate and boreal coniferous forests and, finally, of evergreen and deciduous boreal forests.

## 2 DATA AND METHODS

### 2.1 Pollen dataset

In order to investigate vegetation variability over the Holocene, we consider a large recently compiled dataset of 2802 palynological records extracted from the Neotoma Paleoecology Database and additional literature (Herzschuh et al., 2022) with revised chronologies (Li et al., 2021). The dataset comprised 977 different taxa after taxonomic harmonization of the pollen counts. In order to avoid the drastic post‐deglaciation vegetation changes of the early Holocene, we restrict our analysis to the period starting in 8 ka BP. While it is difficult to completely avoid human impacts, which can go back to the early Holocene in some regions, we do not consider the last 2 ka for the temporal analysis in order to minimize the influence of human‐induced vegetation changes. Therefore, our main analysis includes the period spanning 8–2 ka BP; we also consider the last 500 years of pollen data for the spatial analysis used to establish a biome classification scheme (see Section 2.4). In addition, we restrict the analysis to the records with at least six samples and that are located north of 25°N because of the sparseness of records in the south; we thus retain 1967 records for analysis (Appendix S2: Figure B1). The average mean resolution, largest gaps and temporal coverage of the retained records were, respectively, ~260, ~500, and ~4840 years. Our filtered dataset contains 773 records in North America, 900 records in Eurasia and North Africa west of 60°E, and 294 records in Eurasia east of 60°E. Most records (64%) are located in the mid‐latitude between 40 and 55°N, while we find 14% of them south of 40°N and 22% north of 55°N.

### 2.2 Principal component analysis

To summarize the variability in the multivariate pollen data, there exist several dimension reduction methods that have been used in ecology. We perform what could be described as the simplest dimensional reduction technique: principal component analysis (PCA; Legendre & Legendre, 2012). PCA is a linear method which consists of rotating the coordinate system in order to maximize the variance explained along fewer dimensions. In the case of ecological data such as pollen assemblage data, the original dimensions are the different pollen taxa relative abundances. The newly rotated axes are then linear combinations of the original axes. In our case, we had 997 different taxa, that is, 997 dimensions, although most of them are very infrequent (see examples in Appendix S2: Figure B2).

For a given pollen record with n samples and p taxa, the assemblage matrix A (thus with dimension n×p) consists of proportions obtained by dividing the pollen counts of each taxa by the total pollen counts for a given sample, and therefore the rows of the matrix A sum up to 1 by definition. In order to increase the contribution of less frequent taxa and decrease the interdependence of proportion data, we square‐root transform the assemblage matrices before performing the PCA (Birks, 1998). The principal components correspond to the eigenvectors obtained by diagonalization of the (column‐centred) covariance matrix. The eigenvectors can then be projected back onto the assemblage matrix in order to produce the scores time series for each principal component. The first principal component (PC1), that is, the eigenvector explaining the largest variance in the data, explains on average 46±14% (the error corresponds to one standard deviation) of the variance in single records (Appendix S2: Figure B3a), while this dropped to 17±6% for the second component. The best‐correlated taxon can explain on average 86±11% of the variance of the PC1 score time series (Appendix S2: Figure B3b). Therefore, by considering the PC1 score time series, we can perform time series analysis on a single variable that corresponds to the locally dominant component of pollen variability. There is still ecologically meaningful information in the remaining variance, that is, not explained by the PC1, but as it is spread out over many dimensions, it would be more sensitive to noise and require a different (multi‐variate) methodology; thus, we leave this for future work.

### 2.3 Timescale‐dependent estimates of variability

Given the different resolutions and length of the time series, we need methods which can robustly estimate the timescale‐dependence of the variability from limited and irregular data. Based on the method inter‐comparison from Hébert et al. (2021), we use here the first‐order Haar structure function (HSF) in order to estimate the variability as a function of timescale as its interpolation‐free algorithm is most robust to irregularity for shorter timescales near the resolution of the series. Haar fluctuations at a given timescale τ are given as the average of the absolute difference between the first and second half of temporal intervals of width τ (Haar, 1910; Lovejoy & Schertzer, 2012). The analysis is thus performed in real space (as opposed to Fourier space) and the fluctuations thus have the same units as the input time series; in the case of this study, the compositional changes are given as (unitless) square‐rooted proportions. The confidence intervals are based on the distribution of the estimates from different records available at each timescale. We provide a measure similar to the standard error by dividing the 68% quantile around the mean by the square root of the number of estimates n. To account for the spatial correlation and the limited spatial degrees of freedom in environmental datasets, we limit n to $n_{\max}=100$ for this calculation.

To evaluate the scaling of variability with timescale τ, we assume the power‐law scaling behaviour often observed in geophysical time series (Cannon & Mandelbrot, 1984; Corral & González, 2019; Fedi, 2016; Lovejoy & Schertzer, 1986; Malamud & Turcotte, 1999; Pelletier & Turcotte, 1999) such that for a timescale‐dependent metric $S\left(\tau \right)$ and a general power‐law scaling exponent a the following is approximately true:

(1) $$S\left(\tau \right)\propto \tau^{a}$$

If $S\left(\tau \right)$ is an estimate of the power spectrum, then the exponent a is the scaling exponent traditionally known as β, whereas if $S\left(\tau \right)$ is an estimate of the HSF, then the exponent a corresponds to the fluctuations exponent H; the two can be related by the approximate (or exact in the case of Gaussian processes) relation β≈1+2H (Hébert et al., 2021; Lovejoy & Schertzer, 2012). Therefore, a flat white noise‐like behaviour of β=0 corresponds to H=−0.5, reflecting the fact that averaging white noise over n points decreases its amplitude by a factor of $\sqrt{n}$ and a so‐called “1/f noise”, that is, when β=1, corresponds to H=0. This value H=0 is particularly important as it corresponds to the transition between stationary processes, which converge to a well‐defined mean when H<0, and non‐stationary processes, when H>0 (Lovejoy & Schertzer, 2012). In the case of vegetation, the latter case would thus indicate a regime where the vegetation composition does not oscillate around a stable state, but rather that a shift from one type of vegetation to another is occurring.

We derive three measures of variability from the HSF $S\left(\tau \right)$: the centennial variability $S_{C}$ (mean of $S\left(\tau \right)$ over $\tau \in \left[50,\;200\right]\;\text{years}$), the millennial variability $S_{M}$ (mean of $S\left(\tau \right)$ over $\tau \in \left[500,\;2000\right]\;\text{years}$) and the centennial to millennial scaling $H_{C-M}$ (the fluctuations exponent H fitted over $\tau \in \left[200,\;3000\right]\;\text{years}$). The robustness of the (interpolation‐free) algorithm to estimate the HSF allows us to fit the exponent H over a wide range of timescales with minimal bias, and we use the same method as in Hébert et al. (2021) to fit H, namely a generalized linear model with a gamma distribution model.

### 2.4 Biome classification

In order to identify biomes from pollen assemblages, we consider two standard classification systems to classify recent pollen samples and define so‐called *typical assemblages*. The first classification system we consider is the Olson biome classes (Olson et al., 1985; Appendix S2: Figure B4b), a landmark work based on worldwide inventory systems culminating 20 years of field investigations, consultations and analysis of published literature (Gibbs, 2006). The Olson classes correspond to well‐known biomes such as, for example, Boreal Forests, Temperate Broadleaf Forests, Temperate Coniferous Forest, Grasslands and Tundra (see Appendix S1: Table A1 for the complete list and acronyms). The second classification system we consider is the European Space Agency Climate Change Initiative Land‐Cover (CCI) product, which is based on recent satellite data (ESA, 2017; Appendix S2: Figure B4c). Consequently, the CCI classes are generally related to observable vegetation characteristics such as, for example, whether there are deciduous or evergreen needleleaf trees, broadleaf trees, shrubs, grass or crops (see Appendix S1: Table A2 for the complete list and acronyms). We can thus identify the expected vegetation types more precisely by looking at the spatial intersection of the CCI and Olson classes (Appendix S2: Figure B4a; hereafter we refer to those as *Intersection Classes*). For example, for the numerous records located in areas expected to belong to the Olson class *Temperate Broadleaf & Mixed Forests* (TeBMF), a more accurate separation can be made according to the CCI classes *Broadleaf Deciduous* (BrDec), *Needleleaved Evergreen* (NlEvr) and *Mixed Leaf Type* (MxdLf) and we can also discard those belonging to the *Cropland* (CropL) and *Mosaic Cropland* (MosCr) CCI classes in order to minimize human impacts on the typical assemblages.

The recent pollen assemblages are extracted from the pollen dataset (Section 2.1) by averaging over all pollen samples, for a given record, dated to the period between 500 years BP and the present. The choice to discard the CCI classes disturbed by human agriculture (CropL and MosCr) might thus be overly conservative since the CCI classes are based on satellite data from the last decade. For a given custom‐defined biome, we select one or more intersection classes and define the typical assemblage of the biome as the average of the square‐rooted assemblage of all the recent pollen assemblages belonging to the given intersection classes (for example Appendix S2: Figure B4a). A typical assemblage $a_{i}$ (for a biome i) can then be projected onto the pollen samples from the past, similarly to how the PC1 was projected onto the data, in order to obtain a score time series $u_{i}\left(t\right)$.

However, it is sometimes difficult to differentiate two biomes solely based on these biome scores since there are often taxa common to more than one biome that may contribute significantly to several typical assemblages. For example, *Pinus* was found in the typical assemblages of almost every biomes and will thus have a spurious effect on biome scores (see Appendix S2: Figure B6 for a summary of the typical assemblages defined in this study). We approach this challenge by restricting our analysis to binary comparisons between pairs of biomes, which allows us to define a comparative axis $a_{i,j}$ between the typical assemblages $a_{i}$ of a biome i with respect to that of another biome j such that:

(2) $$a_{i,j}=a_{i}-a_{j}$$

For a given $a_{i,j}$, the associated scores $u_{i,j}\left(t\right)$ that are positive are thus more similar to the first selected biome i, while negative $u_{i,j}\left(t\right)$ are more similar to the biome j. In this work, we only interpret the average of the score time series $u_{i,j}\left(t\right)$ over the 8–2 ka BP time period and denote this mean score by ${\bar{u}}_{i,j}$. For example, in Section 3.2 below, we define a typical forest assemblage $a_{\mathrm{Fo}}$ that we want to compare to the typical assemblage for open‐land $a_{\mathrm{Op}}$ (comprising mainly grassland, tundra and desert, see Appendix S2: Figure B5a). Using this method, we can thus define the comparative axis $a_{\mathrm{Fo},\mathrm{Op}}$, which better differentiates between the two types of vegetation when projected on fossil pollen data. Positive scores along the $a_{\mathrm{Fo},\mathrm{Op}}$ axis thus directly indicate high proportions of forest pollen taxa, while negative scores reflect a high proportion of open‐land pollen taxa. We use this criterion when we iterate the analysis on a subset of the data; for example, we keep the records with ${\bar{u}}_{\mathrm{Fo},\mathrm{Op}}>0$ for an analysis of forest types and discriminate records dominated by open‐land types. See Table 1 for a list of the mathematical symbols used in this study.

**TABLE 1:** Mathematical symbols used to define typical assemblages, that is, the average proportions for all the recent assemblages identified to the given biome, mean scores and measures of variability.

Index	Short name	Long name
1	$a_{\mathrm{Fo}}$	Typical Forest Assemblage
2	$a_{\mathrm{Op}}$	Typical Open‐Land Assemblage
3	$a_{\mathrm{Ne}}$	Typical Needleleaf Forest Assemblage
4	$a_{\mathrm{Br}}$	Typical Broadleaf Forest Assemblage
5	$a_{\mathrm{Bo}}$	Typical Boreal Forest Assemblage
6	$a_{\mathrm{Tc}}$	Typical Temperate Coniferous Forest Assemblage
7	$a_{\mathrm{Be}}$	Typical Boreal Evergreen Forest Assemblage
8	$a_{\mathrm{Bd}}$	Typical Boreal Deciduous Forest Assemblage
9	$a_{\mathrm{Fo},\mathrm{Op}}$	Difference Axis between $a_{\mathrm{Fo}}$ and $a_{\mathrm{Op}}$
10	$a_{\mathrm{Ne},\mathrm{Br}}$	Difference Axis between $a_{\mathrm{Ne}}$ and $a_{\mathrm{Br}}$
11	$a_{\mathrm{Bo},\mathrm{Tc}}$	Difference Axis between $a_{\mathrm{Bo}}$ and $a_{\mathrm{Tc}}$
12	$a_{\mathrm{Be},\mathrm{Bd}}$	Difference Axis between $a_{\mathrm{Be}}$ and $a_{\mathrm{Bd}}$
13	${\bar{u}}_{\mathrm{Fo},\mathrm{Op}}$	Mean score over 8–2 ka BP along $a_{\mathrm{Fo},\mathrm{Op}}$
14	${\bar{u}}_{\mathrm{Ne},\mathrm{Br}}$	Mean score over 8–2 ka BP along $a_{\mathrm{Ne},\mathrm{Br}}$
15	${\bar{u}}_{\mathrm{Bo},\mathrm{Tc}}$	Mean score over 8–2 ka BP along $a_{\mathrm{Bo},\mathrm{Tc}}$
16	${\bar{u}}_{\mathrm{Be},\mathrm{Bd}}$	Mean score over 8–2 ka BP along $a_{\mathrm{Be},\mathrm{Bd}}$
17	$S_{C}$	Mean of Haar fluctuations over $\tau \in \left[50,\;200\right]\;\text{years}$
18	$S_{M}$	Mean of Haar fluctuations over $\tau \in \left[500,\;2000\right]\;\text{years}$
19	$H_{C-M}$	Haar's fluctuations exponent H fitted over $\tau \in \left[200,\;3000\right]\;\text{years}$

## 3 RESULTS

### 3.1 General vegetation variability analysis

**FIGURE 1:**
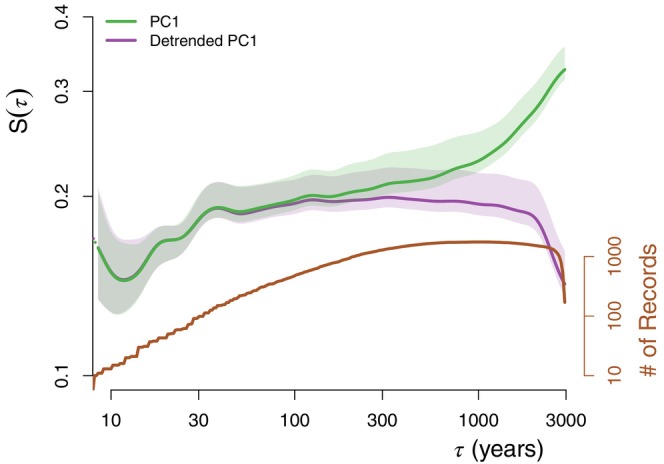
The average first‐order Haar structure function of all the PC1 time series is shown for the undetrended series (green) and the series detrended with a 23‐ka sinusoidal function (purple). The confidence intervals are similar to the standard error, that is, the 68% quantile divided by $\sqrt{n}$ where *n* is the number of records and limited to 100. The number of pollen records able to provide fluctuation estimates at each timescale is indicated by the axis on the right‐hand side (brown).

First, we analyse the general behaviour of vegetation variability irrespective of biome classifications. We computed the HSF from the PC1 of single records and then averaged them together to obtain the average HSF (Figure 1). We find a scaling of variability H≈0.2 over the 300–3000 years timescale band (i.e. H was fitted over $\tau \in \left[300,\;3000\right]\;\text{years}$), while over the 100–1000 years timescale band (i.e. $\tau \in \left[100,\;1000\right]\;\text{years}$), the mean Haar structure function is closer to H=0. We also observe a steepening (with H>0) for τ>1000years and one for τ<100years. If we remove a sinusoidal with a period of 23‐ka years, that is, approximately the period of the orbital precession, then the steepening above the 1000‐year timescale (τ>1000years) disappears such that we have H≈0 over a wide range of scales, and a sudden drop above τ=2000years, which likely indicates that the method is over‐detrending, especially for series with few data points as both the amplitude and phase are allowed to vary. The marked steepening for τ<40years may be related to a characteristic response time of the vegetation, that is, a delay in the vegetation response when environmental conditions change, but it also relies on fewer records and is more uncertain.

**FIGURE 2:**
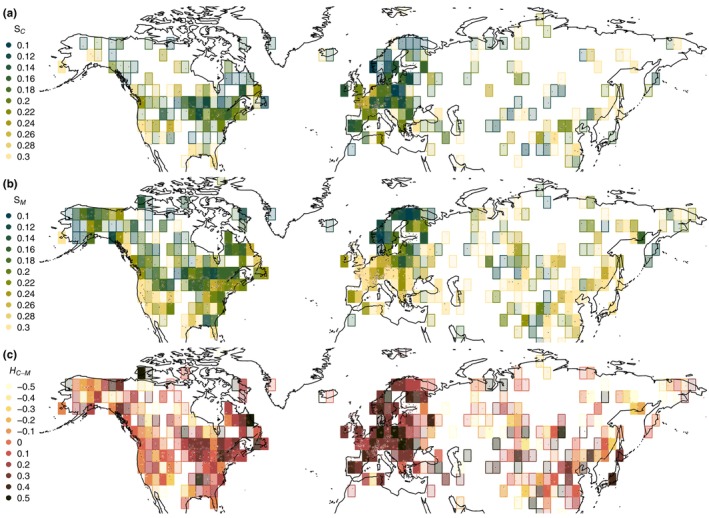
Maps of centennial to millennial vegetation variability. (a) The centennial variability $S_{C}$, that is, the average of the Haar structure functions over the 50–200 years timescale band, is shown for the PC1 time series at the regional scale. The individual records (points) were gridded into 4° by 4° boxes; the opacity of the boxes is linearly proportional to number of records and saturates for 5 records or more. (b) Same as a, but for the millennial variability $S_{M}$, that is, the average of the Haar structure functions over the 500–2000 years timescale band. (c) Same as a, but for the centennial to millennial fluctuations exponent $H_{C-M}$, that is, fitted over the timescale band 100–3000 years.

Using the HSF from the individual records we estimate the spatial patterns of centennial vegetation variability $S_{C}$ (942 series have high enough resolution to provide an estimate), of millennial vegetation variability $S_{M}$ and of centennial to millennial scaling according to the fluctuations exponent $H_{C-M}$ (Figure 2). Quantifying the spatial coherency using Moran's I (Gittleman & Kot, 1990; Paradis et al., 2004) shows that all three metrics are significantly spatially coherent (p<.001). At the centennial timescale, $S_{C}$ displays low variability over Europe, Eastern North America and Northwestern North America, while high variability characterizes most of Asia and Southwestern North America. Similar patterns are observed in $S_{M}$ at the millennial timescales as $S_{M}$ and $S_{C}$ are well correlated (r=.5, p<.01). A notable exception is Europe outside of Fennoscandia which becomes a region of relatively higher variability at millennial timescales, a transition reflected by the higher scaling exponent $H_{C-M}$ found in the continent.

### 3.2 Comparison of forested and open‐land vegetations

**FIGURE 3:**
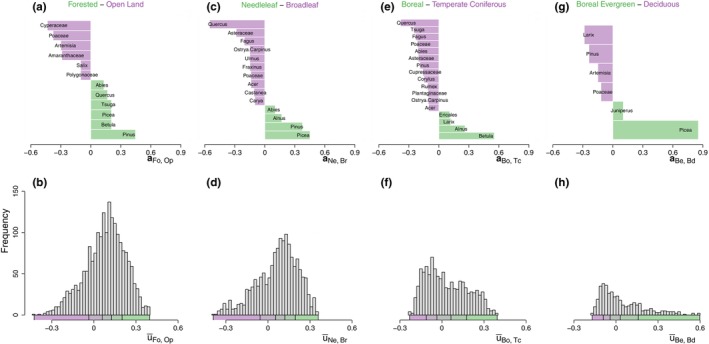
Difference axis and distributions of mean biome scores. (a, c, e, g) The four difference axis defined for the analysis are shown: $a_{\mathrm{Fo},\mathrm{Op}}$, $a_{\mathrm{Ne},\mathrm{Br}}$, $a_{\mathrm{Bo},\mathrm{Tc}}$ and $a_{\mathrm{Be},\mathrm{Bd}}$. The taxa contributing to each axis are given in ascending order, from the most negative (purple) at the top to the most positive (green) at the bottom. For clarity, only taxa with absolute values greater than 0.05 are displayed. (b, d, f, h) The histogram details the mean scores ${\bar{u}}_{\mathrm{Fo},\mathrm{Op}}$, ${\bar{u}}_{\mathrm{Ne},\mathrm{Br}}$, ${\bar{u}}_{\mathrm{Bo},\mathrm{Tc}}$ and ${\bar{u}}_{\mathrm{Be},\mathrm{Bd}}$ obtained over the 8–2 ka BP period for each pollen record along the difference axis on a, c, e, g, respectively. The sites are divided into five groups with an equal number of sites according to the mean biome scores (20% quantiles, shown in colours under the *x* axis).

In order to relate the spatial patterns of vegetation variability to composition, we perform a comparative analysis of sites based on their proximity to taxa belonging to either forested or open‐land biomes. We define a new axis $a_{\mathrm{Fo},\mathrm{Op}}$ to evaluate the proximity of those two types of biomes, as outlined in Section 2.4, using the sites belonging to appropriate intersection classes between the Olson and CCI classes (see Appendix S2: Figure B5a). The newly defined axis $a_{\mathrm{Fo},\mathrm{Op}}$ (Figure 3a) is then dominated by *Pinus*, *Tsuga*, *Betula*, *Picea*, *Quercus* and *Abies* on the (positive) forested side, while Cyperaceae, Poaceae, *Artemisia* and Amaranthaceae dominate the (negative) open‐land side.

**FIGURE 4:**
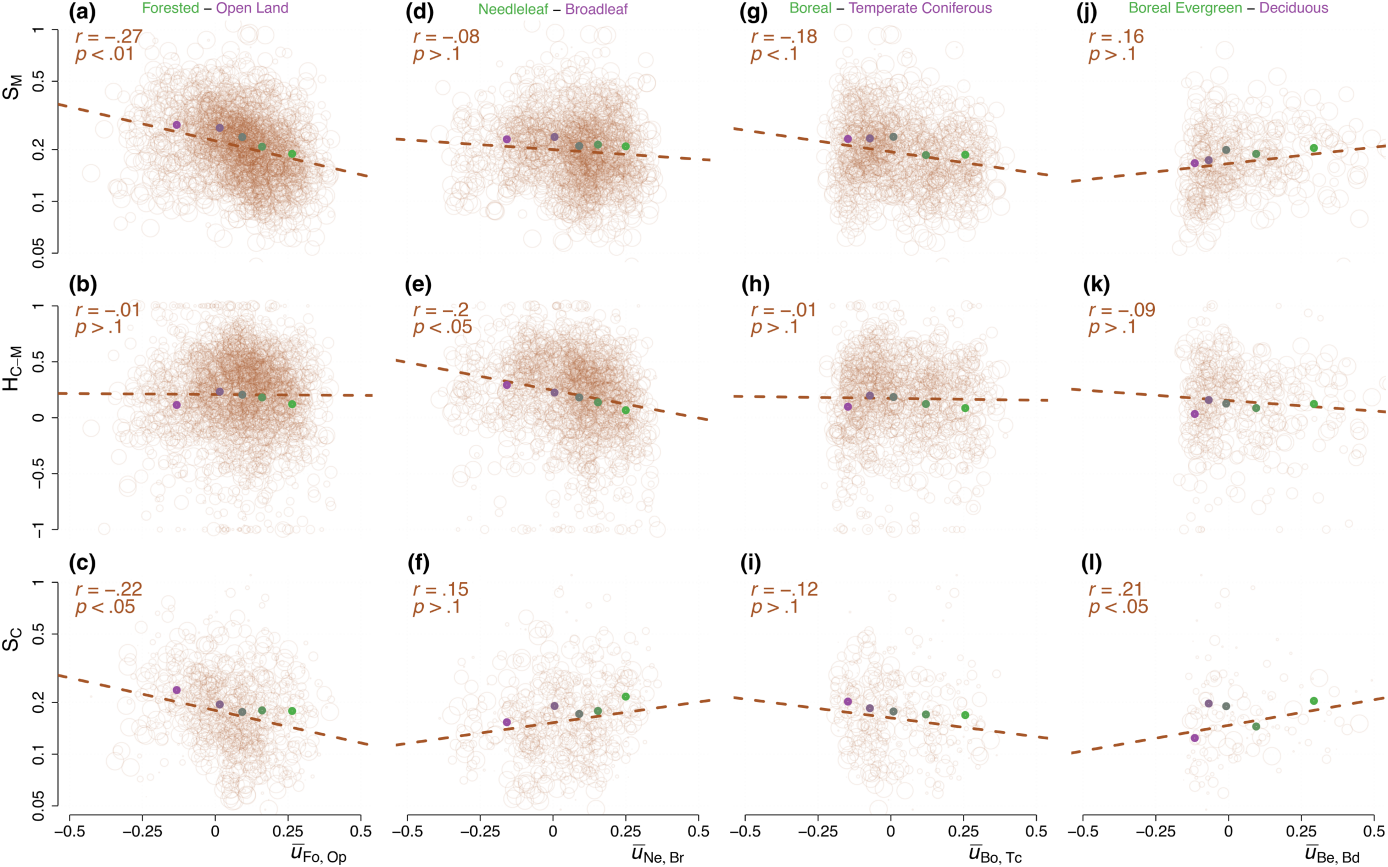
Dependency of the vegetation variability as a function of mean biome scores. (a) The millennial vegetation variability $S_{M}$ is shown as a function of the mean biome score ${\bar{u}}_{\mathrm{Fo},\mathrm{Op}}$. The correlation r and statistical significance *p*‐value p are given in the top left corner; the size of the circles is proportional to the fraction of the timescale band covered (in log), which is also used as weights for the correlation calculation. The average of each 20% quantile is indicated (full circles with colours corresponding to Figure 3b,d,f,h). (b) Same as a, but for the centennial to millennial fluctuations exponent $H_{C-M}$. (c) Same as a, but for the centennial vegetation variability $S_{C}$. (d–f) Same as a, b, c, but as a function of the mean biome scores ${\bar{u}}_{\mathrm{Ne},\mathrm{Br}}$. (g–i) Same as a, b, c, but as a function of the mean biome scores ${\bar{u}}_{\mathrm{Bo},\mathrm{Tc}}$. (j–l) Same as a, b, c, but as a function of the mean biome scores ${\bar{u}}_{\mathrm{Be},\mathrm{Bd}}$.

**FIGURE 5:**
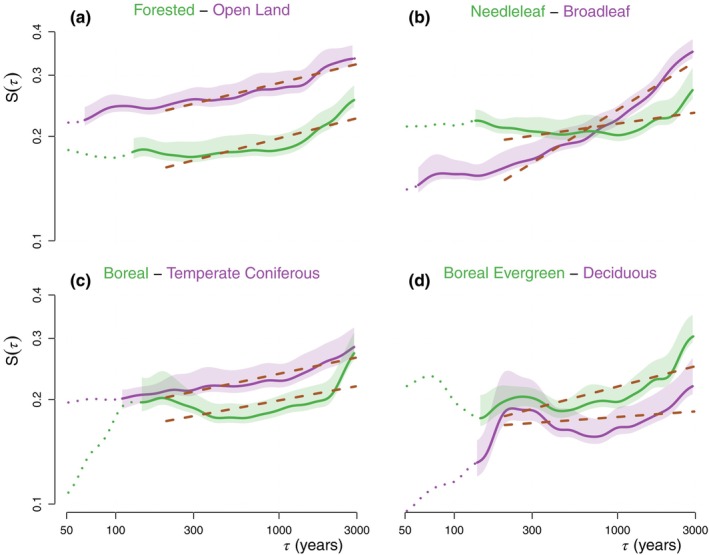
Average HSF of vegetation variability aggregated based on the mean biome scores quantiles. (a) The HSF of the PC1 time series are divided into 20% quantiles according to the mean biome score ${\bar{u}}_{\mathrm{Fo},\mathrm{Op}}$ and averaged together. The lowermost and uppermost quantiles (see Figure 3) are shown with confidence intervals and regression lines (dashed brown) corresponding to $H_{C-M}$. Dotted lines indicate that less than 10 estimates were available at the given timescale. (b) Same as a, but according to ${\bar{u}}_{\mathrm{Ne},\mathrm{Br}}$. (c) Same as a, but according to ${\bar{u}}_{\mathrm{Bo},\mathrm{Tc}}$. (d) Same as a, but according to ${\bar{u}}_{\mathrm{Be},\mathrm{Bd}}$.

The axis $a_{\mathrm{Fo},\mathrm{Op}}$ can then be projected on the pollen data to obtain score time series for each record and the average biome scores ${\bar{u}}_{\mathrm{Fo},\mathrm{Op}}$ are taken over the 8–2 ka BP period. The distribution of mean scores is skewed on the positive side, indicating that forested assemblages dominate the dataset (Figure 3b): there are 1446 positive (forested) mean scores vs 521 negative (open land) ones. The millennial variability $S_{M}$ of the PC1 time series over the corresponding period (8–2 ka BP) is then found to be significantly correlated with the mean biome scores along $a_{\mathrm{Fo},\mathrm{Op}}$ (Figure 4a, r=−.27, p<.01). The same relationship is observed for the centennial‐scale variability $S_{C}$ (Figure 4c, r=−.22, p<.05) indicating that forested assemblages have, on average, significantly lower variability than open‐land ones from centennial to millennial timescales. To clarify the relationship, it is useful to group the sites with similar mean biome scores together. We subdivide the sites into 20% quantiles and compute the average HSF for each quantile (Figure 5a). The result supports the finding of higher variability in open‐land assemblages than in the forested ones (Figure 4a); a flattening of the relationship for the quantiles with negative mean forested biome score seems to indicate little difference in variability between sparse forest and completely open land (Figure 4a).

### 3.3 Comparison of broadleaf and needleleaf forests

To study if the variability depends on the forest type, we identify the sites which were more on the forest side over the period of interest (8–2 ka BP), that is, the 1446 records with ${\bar{u}}_{\mathrm{Fo},\mathrm{Op}}>0$. We perform a new comparison between them along another axis of variability $a_{\mathrm{Ne},\mathrm{Br}}$ opposing broadleaf forests to needleleaf ones (see Appendix S2: Figure B5b for selection of intersection classes). This new axis is dominated by *Picea*, *Pinus*, *Alnus* and *Abies* on the positive (needleleaf) side and by *Quercus*, Asteraceae, *Fagus* and Poaceae on the negative (broadleaf) side (Figure 3c).

The mean scores ${\bar{u}}_{\mathrm{Ne},\mathrm{Br}}>0$ indicate a greater amount of needleleaf sites in the dataset with 1032 positive (needleleaf) ${\bar{u}}_{\mathrm{Ne},\mathrm{Br}}$ and 414 negative (broadleaf) ones (Figure 3d). We again divide these records based on 20% quantiles and compute the average HSF for each 20% quantile (Figure 5b). The relationship between the mean biome score ${\bar{u}}_{\mathrm{Ne},\mathrm{Br}}$ and the PC1 millennial variability $S_{M}$ indicates a weak relationship (r=−.08, p>.1) supporting more stability on the long timescale for needleleaf forests, while the converse is found for $S_{C}$ at centennial timescales (r=.15, p<.1). As a result of this opposite behaviour between centennial and millennial timescales, a stronger relationship (r=−.2, p<.05) is observed between the mean biome score ${\bar{u}}_{\mathrm{Ne},\mathrm{Br}}$ and the centennial to millennial scaling exponent $H_{C-M}$ (Figure 4d–f).

### 3.4 Comparison of temperate and boreal coniferous forests

We iterate a similar analysis as in the previous section on the 1032 records with a needleleaf dominance (i.e. those with ${\bar{u}}_{\mathrm{Ne},\mathrm{Br}}>0$), separating them along the $a_{\mathrm{Bo},\mathrm{Tc}}$ axis opposing boreal forests and temperate coniferous forests. Since there are four times as many surface records of boreal evergreen forests than boreal deciduous forests, the typical boreal assemblage $a_{\mathrm{Bo}}$ tends to be completely dominated by the evergreen assemblage, mainly characterized by *Picea*. In order for $a_{\mathrm{Bo},\mathrm{Tc}}$ to capture both types of boreal forest, we give equal weight to the boreal evergreen and boreal deciduous surface samples when calculating $a_{\mathrm{Bo}}$ (and thus also for $a_{\mathrm{Bo},\mathrm{Tc}}$). On the boreal side of $a_{\mathrm{Bo},\mathrm{Tc}}$ the dominant taxa are then: *Betula*, *Alnus*, *Larix*, Ericales and *Picea*, whereas on the temperate coniferous side they are: *Quercus*, *Tsuga*, *Fagus* and *Abies* (Figure 3e). While it may appear odd that broadleaf taxa are the leading taxa on both sides of the axis, the reason is that *Pinus* dominates both typical assemblages of the boreal and temperate coniferous forests, $a_{\mathrm{Bo}}$ and $a_{\mathrm{Tc}}$, respectively (Appendix S2: Figure B6e,f). The leading contribution of *Pinus* on both axes thus mostly cancels out on the difference axis $a_{\mathrm{Bo},\mathrm{Tc}}$ (Figure 3e). Therefore, when the information on the species level is not available, to identify whether a needleleaf forest dominated by *Pinus* is boreal or temperate, it is more useful to rely on the abundance of secondary broadleaf species occurring concurrently. We thus obtain a statistically significant relationship between ${\bar{u}}_{\mathrm{Bo},\mathrm{Tc}}$ and $S_{H}$ (r=−.18,p<.1), and a similar but weaker one with $S_{C}$ (r=−.12,p>.1) along this axis, indicating higher stability of the boreal forest compared to the temperate coniferous forest over a wide range of timescales (Figure 4g,l).

### 3.5 Comparison of evergreen and deciduous boreal forests

We further refine our analysis to a comparison of boreal forest sub‐types: boreal evergreen forests and boreal deciduous forests. We restrict our analysis to the 413 records contained in the upper two 20% quantiles (i.e. the upper 40% quantile), which thus displayed a clear dominance of boreal forest as this results in a more accurate (and restrictive) selection of sites where boreal forests are present today (Appendix S2: Figure B7). We thus defined a new axis $a_{\mathrm{Be},\mathrm{Bd}}$, mainly determined by *Picea* on the (positive) evergreen side and by *Larix* and *Pinus* on the (negative) deciduous side, and project it on the fossil data to obtain ${\bar{u}}_{\mathrm{Be},\mathrm{Bd}}$ (Figure 3g). We find relationships suggesting that the deciduous boreal forest is more stable than its evergreen counterpart both at the centennial (r=.21, p<.05) and millennial timescales (r=.16, p>.1; Figure 4j,k,l).

**FIGURE 6:**
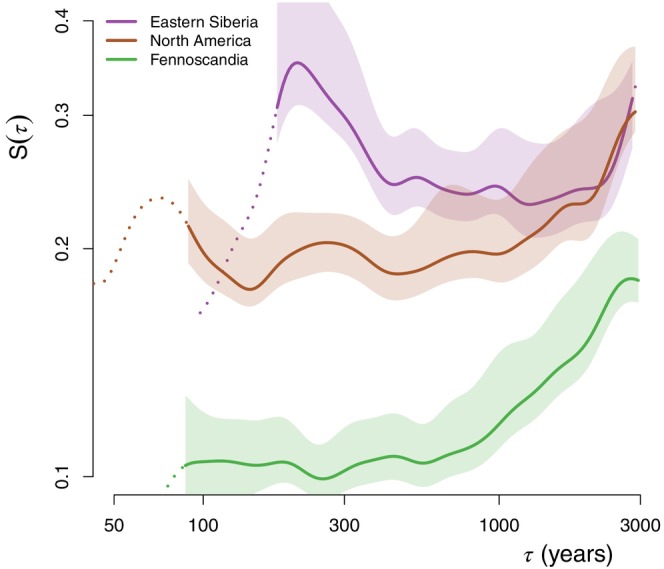
Variability behaviour of boreal forest for three geographical locations. The average HSF of the records belonging to three types of boreal forests are shown: the deciduous forests of Eastern Siberia, the evergreen fennoscandian records and the evergreen records (practically all located) in North America. We used $n_{\max}=20$ for the confidence intervals since the spatial degrees of freedom are reduced.

However, since the boreal evergreen side of the axis is greatly dominated by *Picea*, the Fennoscandian records which are rather dominated by the evergreen taxon *Pinus* are spuriously identified as deciduous (Figure 3g and Appendix S2: Figure B7). As a result, our iterative analysis appears to break down along the $a_{\mathrm{Be},\mathrm{Bd}}$ axis. This can be corrected by comparing the upper and lower 20% quantiles containing the records corresponding most closely to evergreen and deciduous boreal forests, respectively, but separating the lower 20% quantiles according to geographical location into the European records (west of 60°E) and the Asian records (east of 60°E). This results in a different picture which supports a more stable behaviour of the Fennoscandian boreal forest compared to both the *Picea*‐dominated evergreen forest (mostly found in North America in our dataset) and the *Larix*‐dominated deciduous forests of eastern Siberia (Figure 6). There is however a stronger steepening of the HSF for the Fennoscandian records above the 500‐year timescale; H≈0.4 over $\tau \in \left[500,\;3000\right]\;\text{years}$ compared to H≈0.1 for the East Siberian and and H≈0.2 for North American ones.

## 4 DISCUSSION

Our analysis of past vegetation composition inferred from pollen records has revealed strong variability over a wide range of timescales, with fluctuations of similar amplitude from centennial to millennial timescales (H≈0) and of increasing amplitude (H>0) at multi‐millennial timescales, thus indicating long‐term shifts in vegetation composition (Figure 1). The increasing variability towards multi‐millennial timescales is likely forced by the slower trends in orbital insolation and, consequently, it decreases greatly if we detrend the PC1 time series according to a 23‐ka sinusoidal function akin to precession (Figure 1), leading to an extension of the H≈0 scaling behaviour to multi‐millennial timescales. This mirrors results from climate reconstructions, which similarly show H≈0 over multi‐decadal to multi‐millennial timescales (corresponding to a scaling of β≈1 for the power spectrum; Hébert et al., 2022; Laepple & Huybers, 2014b). The scaling of vegetation variability with timescale was found to be strongest in Europe (Figure 2), indicating a non‐stationary behaviour in taxonomic composition. This is consistent with higher rates of post‐glacial vegetation spread in Europe (Giesecke et al., 2017) and translates into stronger millennial‐scale temperature variability in pollen‐based reconstructions (Hébert et al., 2022). This high turnover in Europe might also be influenced by human‐driven changes (Mottl et al., 2021), although we minimized this influence by excluding the last two millennia when the steepest changes in composition occurred.

Open‐land assemblages showed higher variability than their forested counterparts (Figure 5a). Drier climates, characteristic of grassland and other open‐land areas, amplify temperature fluctuations by limiting evaporative cooling based on moisture availability (Sejas et al., 2014). In addition, grasslands are usually characterized by more frequent fire regimes (Leys et al., 2017; Mouillot & Field, 2005), in part due to lower evapotranspiration compared to forests and rapid accumulation of easy‐to‐ignite flammable material (Simpson et al., 2016). It has been argued that fire disturbances play a key role in preserving grassland from forest expansion in areas with high enough precipitation to support forests (Behling et al., 2020; Lasslop et al., 2016; Staver et al., 2011), and consequently periods of decreasing fire disturbances (due to natural causes or human management) have been associated with the expansion of forest into grassland (Grimm, 1983; Portes et al., 2020; Ramezani et al., 2021; Veblen & Markgraf, 1988). The reliance of grassland on fire disturbances to maintain itself could also explain why periods of high vegetation variability have been linked to grassland occurrence (Sobol et al., 2019).

Among the different forest types, broadleaf deciduous forests are the least variable on shorter timescales, but most unstable at longer timescales as evidenced by their stronger scaling (Figure 4e). As such, they exhibit lower variability than their needleleaf counterparts on centennial timescales, but this reverses on millennial timescales (Figure 4d,f). The lower centennial‐scale variability of broadleaf trees may relate to their fire‐suppressing capacities (Feurdean et al., 2017; Kuuluvainen, 2002), while needleleaf trees and their understory are more flammable (Feurdean et al., 2017; Mutch, 1970). The steeper scaling of the broadleaf forest on millennial timescales on the other hand may be the result of post‐glacial changes in eastern North America and western Europe (Davis, 1983) where most of the broadleaf records in our dataset are located (Appendix S2: Figure B7).

While fire disturbances increase variability for shorter timescales, they may act as a stabilizing process for longer timescales by preventing the migration of new species less adapted to fire. Needleleaf taxa such as *Larix* and certain species of *Pinus* have adapted to survive fire episodes with, for example, thicker bark and the shedding of low‐lying branches (Wirth, 2005; Wirth et al., 1999). As such, it has been proposed that fire plays a major role in the dominance of *Larix* in Central Siberia (Rogers et al., 2015; Schulze et al., 2012) and led to a state of vegetation‐climate disequilibrium in warmer areas still forested by *Larix* (Herzschuh et al., 2016). The hypothesis of a stabilizing effect of fire on long timescales is supported by the observed lower variability for needleleaf forest on millennial timescales (Figure 4d), and further reinforced by the analysis of boreal forest (Section 3.5) based on geographical provenance (Figure 6); the records located in Fennoscandia where fire disturbances are less frequent were found to be less variable than those in Eastern Siberia and North America characterized by more active fire regimes (Mouillot & Field, 2005). The gap in variability between Fennoscandia and the other regions was larger on sub‐millennial timescales and almost vanishes at the 3000‐year timescale, that is, the longest timescale investigated where the vegetation response to climate is most important (Webb, 1986). The steep scaling of vegetation variability in Fennoscandia on millennial timescales (Figure 6) may thus be related to a climatic shift towards continentality that occurred over the last 7000 years (Giesecke et al., 2008).

We focused on very broad characteristics to explain the variability and mainly related them to fire vulnerability. This is of course a simplification. In the case of the forests and open‐land comparison, we note that types of open‐land assemblages which are less represented in the dataset such as tundra and deserts might behave differently than grassland. Similarly, in the broadleaf and needleleaf comparison, we note that, among needleleaf species, different fire adaptation strategies exist and that likewise not all broadleaf trees resist well to fire (Saura‐Mas et al., 2010). In addition, for all comparisons, the low taxonomic resolution we had to employ for the study confounds species that may have different behaviours; this is the case for *Pinus* species which can be classified into fire resisters and fire avoiders (Fonda, 2001). These confounding factors may explain the high dispersion of variability estimates as a function of biome scores (Figure 5). Furthermore, the effect of fire, climate and vegetation feedback are intrinsically interconnected (Harris et al., 2016): dry (moist) climates contribute to more (less) fire disturbances and compounded higher (lower) vegetation variability, and, therefore, what was explained in terms of fire regime above could also be related to climate feedback. Finally, pollen assemblages do not directly reflect past vegetation composition as there are biases in pollen accumulation due to different dispersal characteristics and pollen productivity (Theuerkauf & Couwenberg, 2018). While efforts have been made to take those challenges into account to estimate past vegetation cover (Sugita, 2007), it remains challenging to do so given our limited knowledge of pollen productivity estimates and fall speed (Wieczorek & Herzschuh, 2020). The extent of the accumulation basin also impacts the spatial scale of the recorded signal as a wide basin will average over a larger spatial area and reduce local effects.

## 5 CONCLUSIONS

We have analysed the variability of past vegetation composition inferred from pollen records and found increasing variability with increasing timescale, as well as spatially coherent patterns in vegetation variability over centennial to millennial timescales. In addition, significant relationships were identified between the vegetation variability over those timescales and the mean vegetation composition, based on binary comparisons between broadly defined biomes. High natural variability was linked to open‐land and needleleaf biomes with more active fire regimes, as opposed to forested and broadleaf biomes, respectively. Paradoxically, this higher variability was often concurrent with weaker scaling and thus appeared to stabilize the vegetation composition on the longer timescales and could reduce climate‐induced long‐term shifts. However, it remains challenging to study the vegetation climate link over the Holocene since pollen data generally provides the basis for both climate and vegetation reconstructions, and thus they cannot be reconstructed independently. In future studies, evaluating the vegetation response to orbital insolation and other climate forcing with a mechanistic model would allow a better separation of internal and forced response. In addition, large‐scale multi‐proxy studies involving independent climate and fire archives coming from nearby lakes and marine sediments should be undertaken to better elucidate the attribution of vegetation variability to fire disturbances.

## AUTHOR CONTRIBUTIONS

**Raphaël Hébert:** Conceptualization (lead); formal analysis (lead); methodology (lead); software (lead); writing – original draft (lead); writing – review and editing (equal). **Laura Schild:** Validation (lead); writing – review and editing (equal). **Thomas Laepple:** Conceptualization (supporting); formal analysis (supporting); methodology (supporting); supervision (equal); writing – original draft (supporting); writing – review and editing (equal). **Ulrike Herzschuh:** Conceptualization (supporting); data curation (lead); formal analysis (supporting); supervision (equal); writing – original draft (supporting); writing – review and editing (equal).

## CONFLICT OF INTEREST STATEMENT

The authors declare no competing interests.

## Supporting information


Appendix S1 and S2.

## Data Availability

The code and data are available publicly on Zenodo with the identifier https://doi.org/10.5281/zenodo.8226179. The R package RScaling is available in Zenodo with the identifier https://doi.org/10.5281/zenodo.7062678.
