# Supplementary material for: Biome‐ and timescale‐dependence of Holocene vegetation variability in the Northern Hemisphere
Source: Ecol Evol. 2023 Oct 24;13(10):e10585. doi: 10.1002/ece3.10585 (PMC10598260; doi:10.1002/ece3.10585)
Supplement: Supplementary file 1 — Appendix S1 and S2. [file ECE3-13-e10585-s001.pdf]

Appendix S1 : Supplementary Tables

Table A1. Short names for Olson classes (Olson et al., 1985).

Index	Short Name	Long Name
1	TrMBF	Tropical & Subtropical Moist Broadleaf Forests
2	TrDBF	Tropical & Subtropical Dry Broadleaf Forests
3	TrCoF	Tropical & Subtropical Coniferous Forests
4	TeBMF	Temperate Broadleaf & Mixed Forests
5	TeCoF	Temperate Coniferous Forests
6	BoreF	Boreal Forests
7	TrGSS	Tropical & Subtropical Grasslands, Savannas & Shrublands
8	TeGSS	Temperate Grasslands, Savannas & Shrublands
9	FloGS	Flooded Grasslands & Savannas
10	MonGS	Montane Grasslands & Shrublands
11	Tundra	Tundra
12	MeFWS	Mediterranean Forests, Woodlands & Shrub
13	DesXS	Deserts & Xeric Shrublands

Table A2. Short names for CCI classes (ESA, 2017)

Index	Short Name	Long Name
1	CropL	Croplands
2	MosCr	Mosaic Croplands
3	BrDec	Broadleaved Deciduous
4	NlEvr	Needleleaved Evergreen
5	NlDec	Needleleaved Deciduous
6	MxdLf	Mixed Leaf Type
7	MosTS	Mosaic Tree and Shrub Herbaceous Cover
8	MosHe	Mosaic Herbaceous Cover
9	Shrub	Shrubland
10	Grass	Grassland
11	LichM	Lichens and Mosses
12	SprsV	Sparse Vegetation
13	TFloo	Tree Cover Flooded (Fresh or Brakish Water)
14	SFloo	Shrub or Herbaceous Cover Flooded (Fresh/Saline/Brakish Water)
15	Urban	Urban Areas
16	BareA	Bare Areas
17	SnowI	Snow and Ice

Appendix S2 : Supplementary Figures

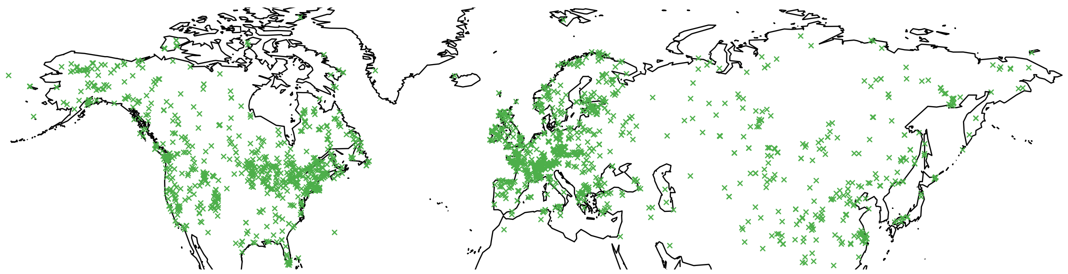

Figure B1. Locations of the 1967 palynological records analysed.

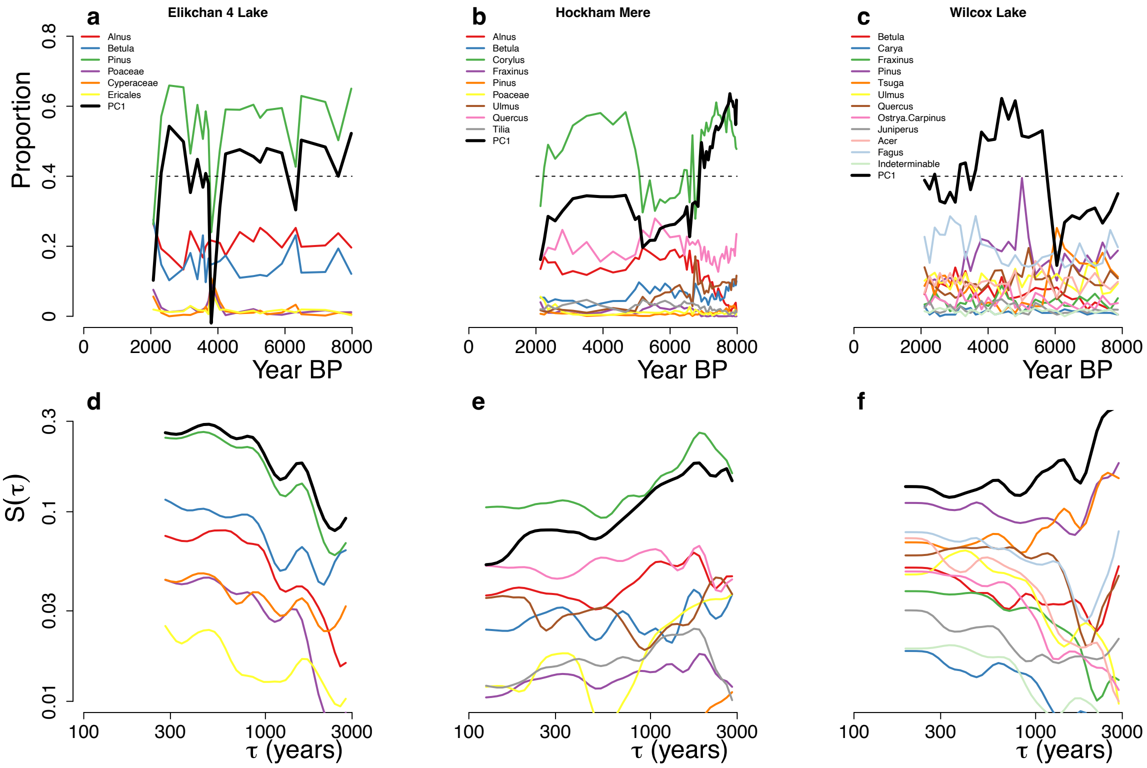

Figure B2. Examples of taxa and PC1 timeseries, and their HSF. **a,b,c** Examples of the taxa timeseries present in four cores extracted from the dataset. Only taxa which had an average proportion of at least 1% are shown. The PC1 timeseries is indicated along (thick black), but shifted up by 0.4 so that it oscillates around the dashed line. **d,e,f** The HSF of the timeseries above are shown, i.e. the average fluctuation amplitude as a function of timescale.

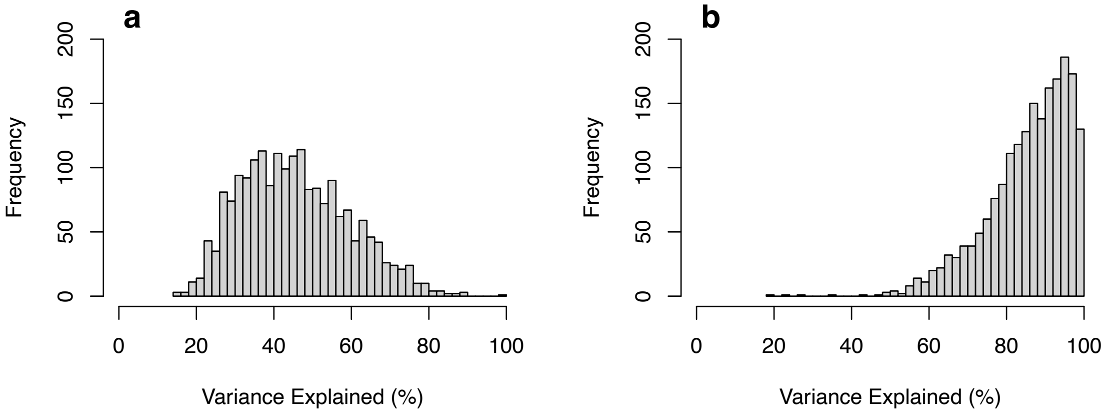

Figure B3. Distributions of variance explained by the PC1 and leading taxa. **a** Percentage of the variance of the assemblage matrix explained by the PC1. **b** Percentage of the variance of the PC1 score timeseries which can be explained by a single taxon. Thus, for each record, we calculated the amount of the PC1 variance explained by each taxa and reported the maximum value.

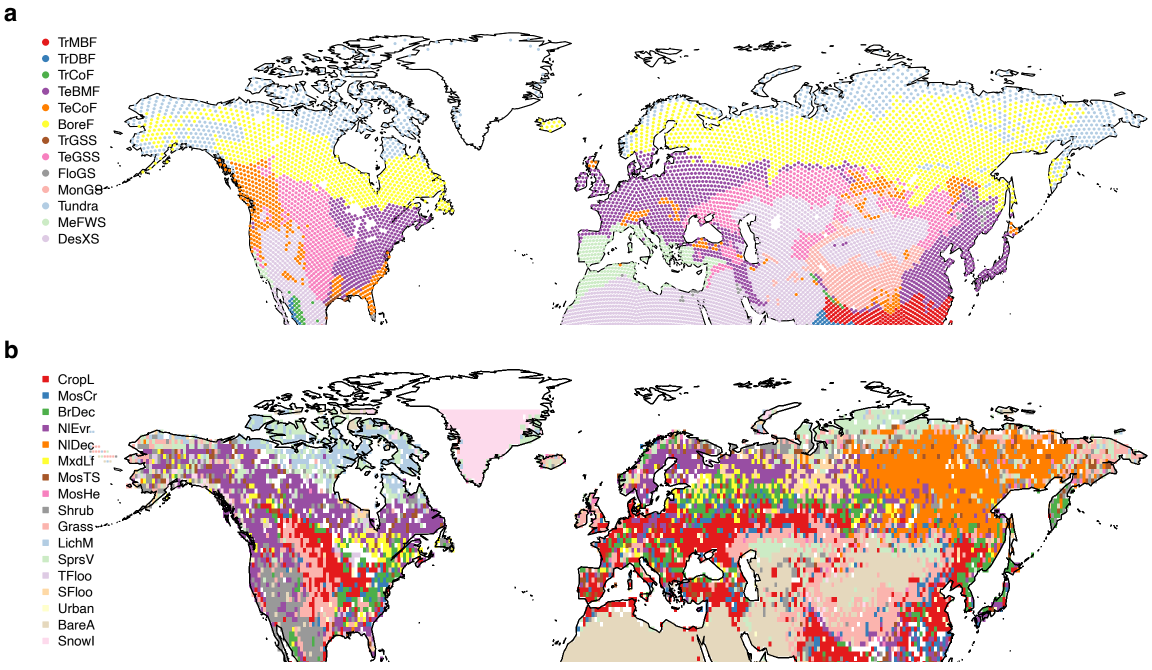

Figure B4. a Map of the areas belonging to each Olson classes, and **b** CCI classes. See Tables A1, A2 for the lists of acronyms used in the legends.

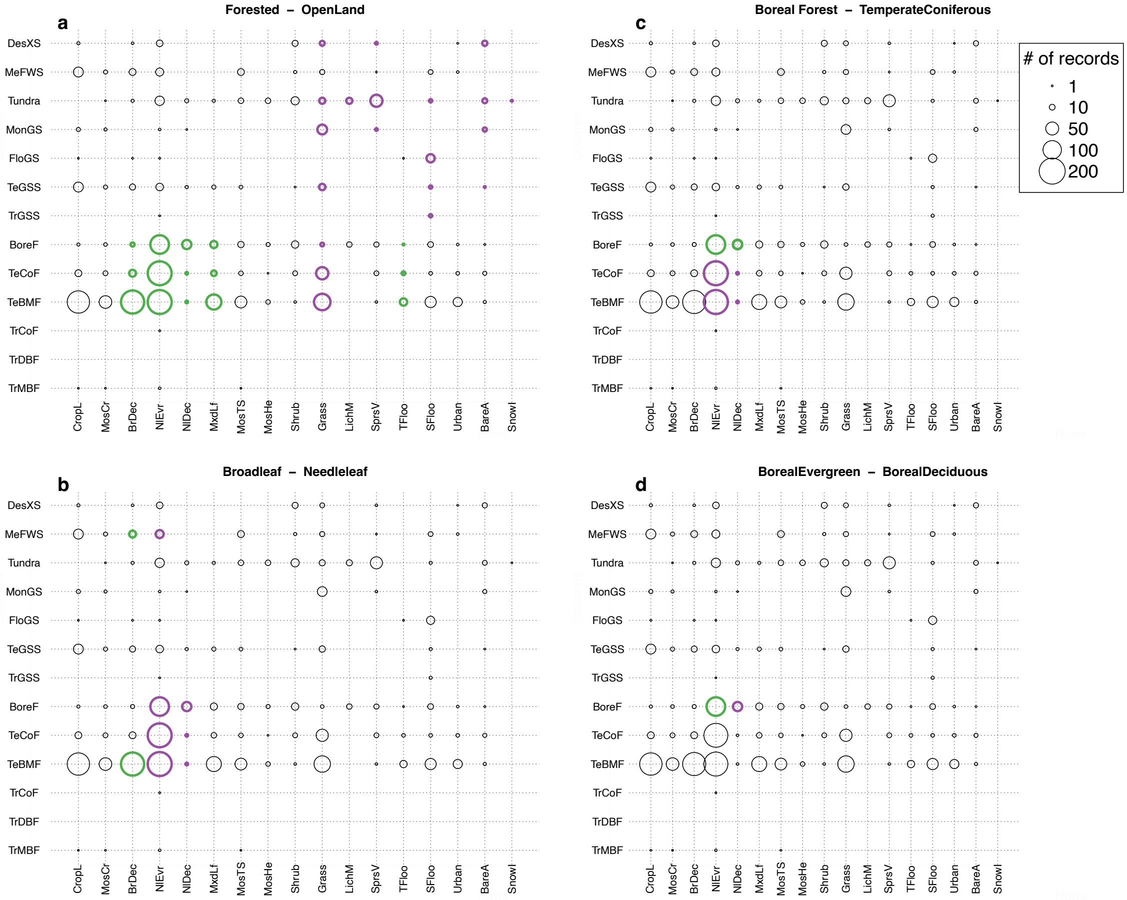

Figure B5. The intersection classes selected to define the typical assemblages used to define the binary axis (see section 2.4) are indicated in green, for those defining the typical assemblage on the positive side, and in purple, for those defining the typical assemblage on the negative side. The area of the circles is proportional to the number of recent samples identified to the given intersection class (as indicated by the legend). The panels indicate the selection for each of the binary comparisons considered in the main analysis: **a.** $\boldsymbol{a}_{\mathrm{Fo,Op}}$, **b.** $\boldsymbol{a}_{\mathrm{Ne,Br}}$, **c.** $\boldsymbol{a}_{\mathrm{Bo,Tc}}$, **d.** $\boldsymbol{a}_{\mathrm{Be,Bd}}$.

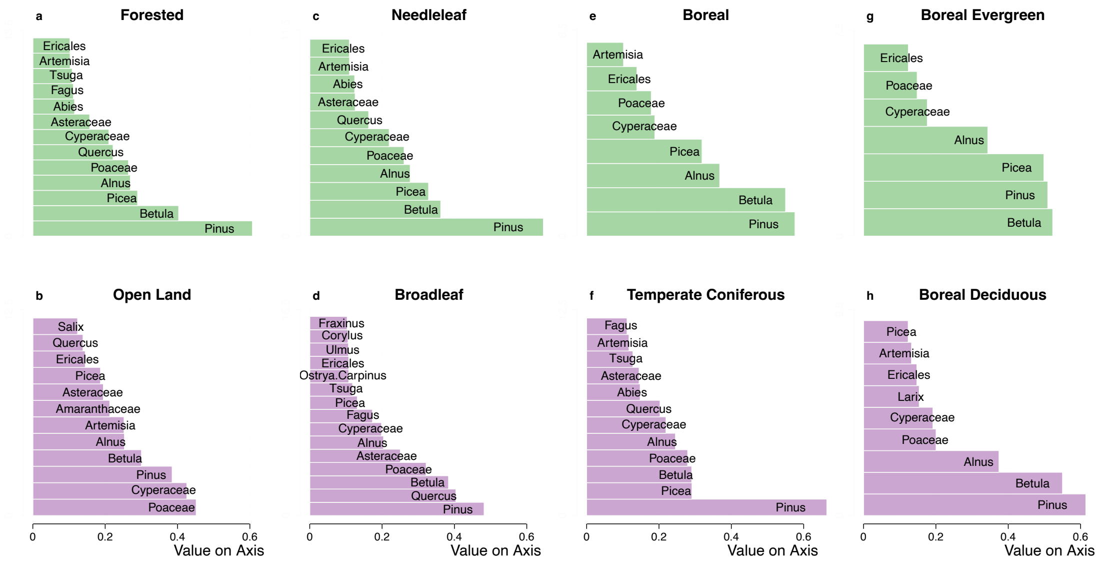

Figure B6. The typical assemblages shown are **a.** $\boldsymbol{a}_{\mathrm{Fo}}$, **b.** $\boldsymbol{a}_{\mathrm{Op}}$, **c.** $\boldsymbol{a}_{\mathrm{Ne}}$, **d.** $\boldsymbol{a}_{\mathrm{Br}}$, **e.** $\boldsymbol{a}_{\mathrm{Bo}}$,**f.** $\boldsymbol{a}_{\mathrm{Tc}}$, **g.** $\boldsymbol{a}_{\mathrm{Be}}$ and **h.** $\boldsymbol{a}_{\mathrm{Bd}}$ (see Table 1 for list of definitons). They were defined as the average of the square-rooted recent pollen samples belonging to the corresponding intersection classes as shown on Fig. B5. See section 2.4.

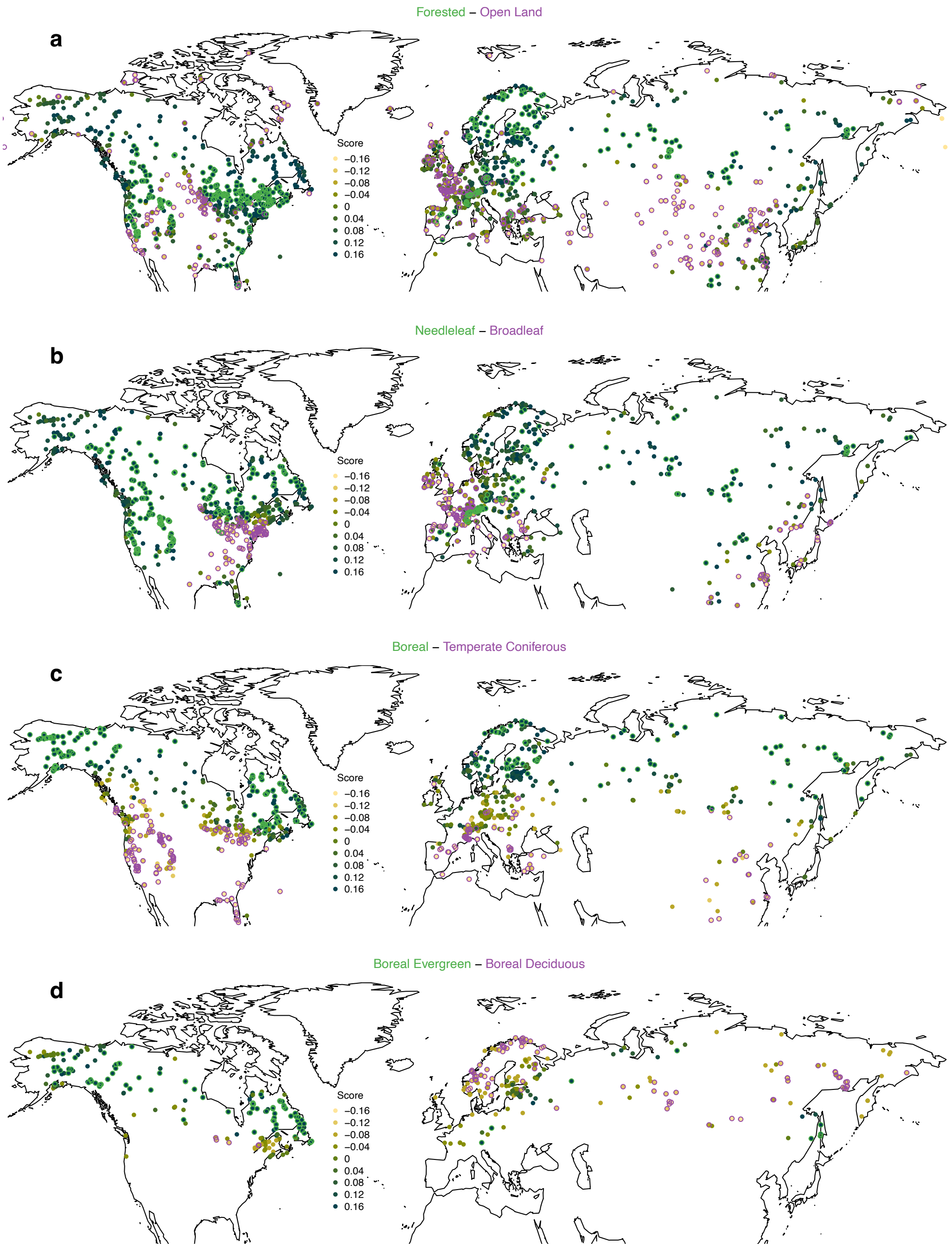

Figure B7. Maps of the mean biome scores for each binary analysis, thus also indicating which records were kept. The records belonging to the uppermost and lower 20% quantiles are indicated by purple and green edged markers respectively; the quantiles are the same as those shown in the main analysis.
